# Editorial: Psychosomatic Aspects of Obesity

**DOI:** 10.3389/fpsyt.2020.614903

**Published:** 2020-11-10

**Authors:** Melissa Ann Kalarchian, Martina De Zwaan

**Affiliations:** ^1^School of Nursing, Duquesne University, Pittsburgh, PA, United States; ^2^Hannover Medical School, Hanover, Germany

**Keywords:** psychosomatic, bariatric surgery, stigma, cognitive science, renal transplant, obesity

The prevalence of obesity has increased worldwide to pandemic levels (1). Obesity-related comorbidities can include diabetes, heart disease, and certain types of cancer, as well as depression and eating disorders. Characterized by excess energy intake over expenditure, obesity is a heterogeneous condition with diverse causal and maintaining factors (2). Although there is no cure for obesity, it can be managed with behavioral, pharmacological, and surgical approaches. Given the challenges of treating obesity, there has been increasing emphasis on individualized approaches to prevention and early intervention (3).

This Research Topic includes 7 contributing articles that address various psychosomatic aspects of obesity. Bayer et al. conducted a telephone survey in Germany regarding perceptions of responsibility for maintaining a healthy diet and body weight. Most respondents indicated that responsibility lies with the individual, suggesting a tendency to underestimate the contribution of societal and environmental factors in obesity. This is particularly important given a growing appreciation that public health messages should be based on accurate information about obesity in order to avoid stigma and discrimination (4).

Several articles in this Research Topic touch on obesity and the interplay between impulsivity, inhibition, and reward sensitivity. Naets et al. report on maladaptive eating in children and adolescents with moderate to extreme obesity, evaluating differences in inhibition among 572 participants in a Belgian inpatient treatment center. Results of hierarchical linear regression models suggest that the role of inhibition in weight loss varies by age and gender, highlighting the importance of identifying subgroups for tailoring interventions. Schag et al. also examined differences in inhibition in a pilot study conducted in Germany comparing patients with alcohol use disorder (AUD) to patients with binge eating disorder (BED). Results indicate that patients with AUD and BED process disorder-specific stimuli (alcohol and food, respectively) differently as compared to healthy controls and neutral stimuli. Findings address the ongoing debate surrounding the utility of extending the concept of addiction to the treatment of obesity and eating disorders (5, 6).

Schäfer et al. also draw on the field of addiction research, examining decision making with the Cards and Lottery Task, documenting good reliability and validity among individuals in Germany with severe obesity, some of whom were scheduled for bariatric surgery. Lescher et al. also studied decision-making, as measured with a modified version of the Iowa Gambling Task, in bariatric surgery patients as compared to people without obesity in Germany. Food pictures interfered with decisions in both groups, but not in the ways that were predicted. Results of these studies highlight the challenges and opportunities for drawing on the latest findings in cognitive science to develop robust conceptual frameworks, research paradigms, and interventions that can translate to the real world outside of the laboratory setting (7).

Robitzsch et al. assessed bariatric surgery patients in Germany, considering not only deficits but also psychological resources. Path analyses indicated that depression and eating behavior mediated the relationship between psychological resources and obesity in 127 candidates for surgery. Findings may inform efforts to identify individuals at risk and deliver individualized supports in the service of improving outcomes after bariatric surgery (8).

Finally, it is important to keep in mind that obesity is associated with certain medications and medical conditions. In this Research Topic, Nöhre et al. examined obesity after kidney transplantation among 433 patients in a German post-transplant care program, documenting an association between increasing body mass index and decreasing graft functioning. Rates of post-transplant obesity varied by age and gender. Results again underscore the need for specialized approaches to weight management in vulnerable groups (9).

Collectively, the 7 contributing articles address the psychosomatic aspects of obesity across the lifespan, utilizing a variety of research designs including participants with and without obesity, recruited from community and clinical settings. Although none of the studies reported here are clinical trials, all of them can inform efforts at prevention and intervention that are tailored to the unique needs of individuals who are vulnerable to obesity. This is particularly important as obesity is increasingly recognized as a chronic health condition and a growing public health problem worldwide. Interdisciplinary, multicomponent interventions are needed at the individual, community, and policy levels (10). We hope that this Research Topic will stimulate further work in this important area.

## Author Contributions

MK wrote the article. MD critically revised and approved the final version of the manuscript.

## Conflict of Interest

The authors declare that the research was conducted in the absence of any commercial or financial relationships that could be construed as a potential conflict of interest.
